# Prostate cancer treatment in Portugal: a nationwide analysis

**DOI:** 10.1038/s41598-023-46591-1

**Published:** 2023-11-08

**Authors:** Francisco Botelho, Rui Lopes, Francisco Pina, Carlos Silva, Luís Pacheco-Figueiredo, Nuno Lunet

**Affiliations:** 1 Serviço de Urologia do Centro Hospitalar Universitário S. João, Alameda Hernâni Monteiro, 4200-319 Porto, Portugal; 2 Instituto de Investigação em Ciências da Vida e Saúde - ICVS/3B’s Laboratório Associado, Escola de Medicina, Universidade do Minho, Braga, Portugal; 3 Escola de Medicina, Universidade do Minho, Braga, Portugal; 4 Departamento de Urologia, Faculdade de Medicina, Universidade do Porto, Porto, Portugal; 5 Departamento de Ciências da Saúde Pública e Forenses e Educação Médica, Faculdade de Medicina, Universidade do Porto, Porto, Portugal; 6 EPIUnit - Instituto de Saúde Pública, Universidade do Porto, Porto, Portugal

**Keywords:** Prostate cancer, Prostate, Epidemiology

## Abstract

Different treatment options exist for localized prostate cancer. Treatments performed in high-volume hospitals are associated with better results. Our objective was to describe time trends in prostate cancer treatments in Portugal and case volume per hospital. We used the national database of diagnosis-related group of the Portuguese Central Administration of the Health System to describe the number of radical prostatectomy (RP), brachytherapy (BT) and external radiotherapy (eRT) treatments performed in all National Health System hospitals. There was a rapid increase in the annual number of RP until 2006 and then a deceleration; BT treatments augmented significantly until 2011. The utilization of eRT also increased, surpassing RP after 2010. From the 46 hospitals performing RP, only eight had a case-volume > 50 treatments/year, and from the nine hospitals performing BT, only four accomplished > 15 treatments/year. In the 11 hospitals with eRT, nine performed > 50/year. Regarding RP, there was negative correlation between the hospital volume and length of stay (r = − 0.303; p = 0.041). In the Portuguese National Health Service there was a steep increase in the number of prostate cancer treatments, and there is an ample margin for concentration of RP and BT treatments, for improvement of the hospitals case volume.

## Introduction

Prostate cancer is the second most common cancer and the fifth leading cause of cancer death among men worldwide, with an estimated 1,414,259 new cases and 375,304 deaths in 2020^1^.

The standard treatments for localized prostate cancer are radical prostatectomy (RP), external beam radiotherapy (eRT) and brachytherapy (BT). Active surveillance is also an option for patients with low risk diseases^2^. The choice of treatment is always complex and depends on stage, biopsy grade, prostatic specific antigen (PSA) value, age and respective co-morbidities as well as on the patients’ preferences, availability of the treatments, and experience of the urologist.

RP performed in high-volume hospitals is associated with better outcomes including reduced mortality, morbidity, postoperative complications, length of stay and cost^3^. Also for BT, men treated at higher volume hospitals had lower rate of complications^4^. Regarding eRT there is evidence that treatment at centers with higher case volume is associated with improved overall survival and lower rates of secondary therapy^5,6^. Recommendations from an expert group of the European School of Oncology state that prostate cancer patients should be treated in a prostate cancer unit performing more than 50 RP, 50 RT and 15 BT treatments per year^7^.

In Portugal, prostate cancer was estimated to account for 6759 incident cases and 1917 deaths in 2020^1^, making it the most frequent cancer and the third leading cause of oncological death in men. As in most developed countries, the incidence rates have been increasing since 1998, along with declining mortality. To the best of our knowledge, there is no national published data on the patterns of treatments used in localized prostate cancer, and therefore this study aimed to describe time trends in prostate cancer treatments and corresponding case volume in Portuguese public hospitals in the most recent years.

## Results

**Figure 1:**
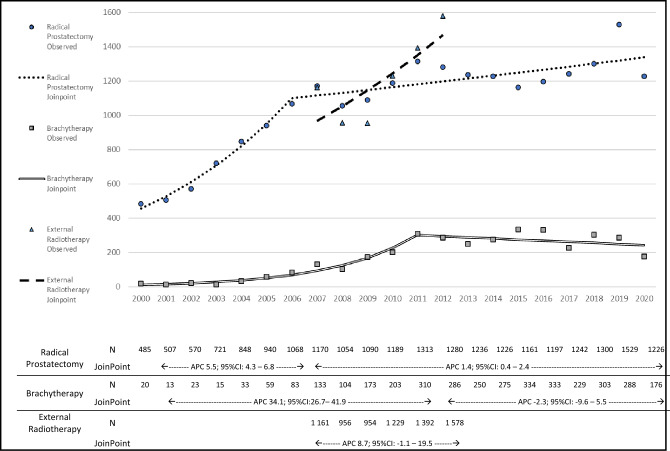
Treatments performed in public hospitals for localized prostate cancer patients, per year, in Portugal, with Joinpoint analysis of the trend. *JoinPoint* JoinPoint analysis results, *APC* annual percent change, *95%CI* 95% confidence intervals; *N* number of procedures per year.

The trends in the use of the different treatments are presented in Fig. 1. Between 2000 and 2020, a total of 21,360 RP and 3610 BT treatments were performed. In the whole period the number of procedures increased 253% for RP and 880% for BT with a peak of 1529 RP in 2019 and 334 BT in 2015.There was a steep increase in the annual number of RP treatments until 2006, corresponding to an APC of 15.9% (95% CI 10.5 to 21.5), and then a less pronounced increasing trend until 2020 (APC: 1.4%; 95% CI 0.5 to 2.4). For BT, the number of treatments increased at an APC of 32.4% (95% CI 23.0 to 42.5) until 2011, and levelled thereafter (APC: − 1.7%; 95% CI − 5.9 to 2.7).

Between 2007 and 2012, a total of 7270 patients were treated with eRT for prostate cancer. During this period there was an increase of 36%, with a peak of 1578 cases in the last year, corresponding to an APC of 8.8% (95% CI − 0.2 to 18.6). After 2010, the number of patients treated with eRT surpassed the number of those submitted to RP. Combining all treatments, in 2012 there were more 42% of patients treated for prostate cancer than in 2010.

The case volumes per hospital in the most recent years is presented in Table 1 but complementary graphics for RP and BT are provided (Supplementary Material). The median number of treatments was 19 for RP, 128 for RT and 14 for BT. From the 46 hospitals performing RP, 14 performed less than 10 treatments/year and eight more than 50 treatments /year; the latter hospitals treated 47.7% of all the patients submitted to this procedure. There was an increase in the proportion of Laparoscopic/robotic RP, from 18.3% in 2017, to 32.8%, in 2020.

**Table 1:** Hospital’s case volume for prostate cancer treatments and their use per region.

	Radical Prostatectomy	Brachytherapy	External Radiotherapy
Case volume per hospital			
Years of analysis	2018–2020	2018–2020	2010–2012
Median	19.3	14.3	128.0
P25-P75	7.8 – 43.8	9.7 – 16.7	66.3 –189.2
N (%) hospitals with adequate case volume*	8 (17.4%)	4 (44.4%)	9 (81.8%)
Case volume per type of hospital (median)^§^			
Hospitals group IV-a	33	86	302
Hospitals group III	75	14	136
Hospitals group I and II	15	1	50
Treatments in 2012 per region (%)			
North	45.9	5.8	48.3
Center	29.4	18.9	51.7
South	42.9	6	51.1

*According to the criteria of the expert group of the European School of Oncology: ≥ 50 RP, 50 RT and 15 BT treatments per year (7).

^§^Hospitals group IV-a, hospitals group III and hospitals group I and II (39) correspond to oncology hospitals, generic central university hospitals, and other generic hospitals, respectively.

Among the nine hospitals performing BT, the average case-volume ranged from 102 treatments/year to less than 1 treatments/year. The two hospitals with the highest volume performed more than 80 treatments/year (treating 73.5% of all the patients undergoing BT during this period) while all the others performed less than 20 treatments/year. There were four hospitals that performed more than the recommended 15 cases/year.

Between 2010 and 2012, eRT was performed in 11 hospitals; the number of treatments was greater than 50/year in nine, from which only two treated more than 300 patients per year each. Hospitals type IV-a performed the highest number of eRT and BT treatment per year (median of 86 and 302, respectively), followed by hospitals type III (median of 14 and 136, respectively) and then hospitals type I and II (median of 1 and 50, respectively). Regarding RP, the highest case volume was in hospitals type III, followed by hospitals type IV-a and then hospitals type I and II (median of 75, 33 and 15, respectively).

The distribution of treatments by region in 2012 is presented in Table 1. In 2012, the proportion of BT treatments was higher in the Center and the use of RP was more frequent in the North (por valores).

**Figure 2:**
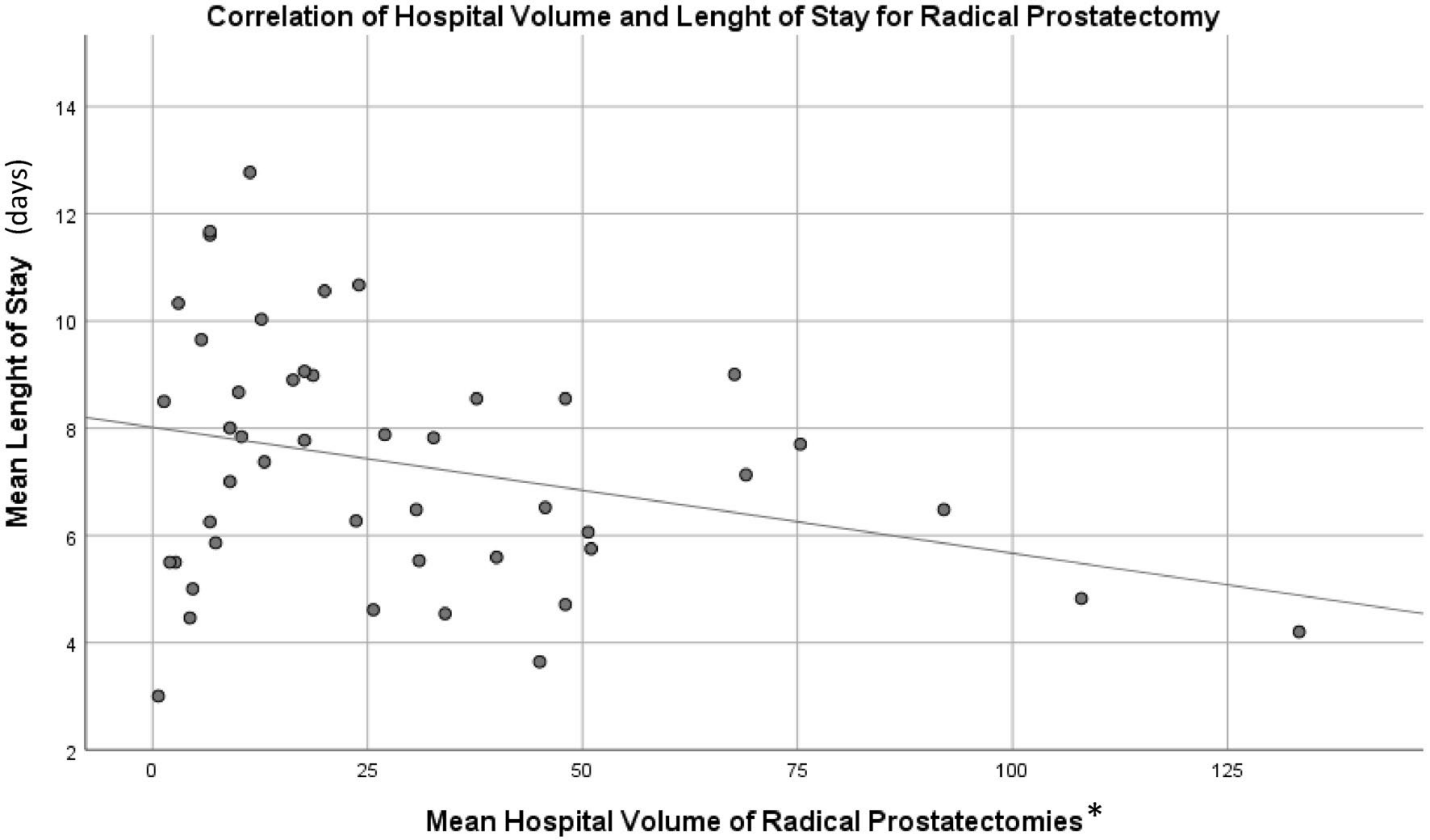
Correlation of hospital volume and length of stay for radical prostatectomy in Portugal (2018–2020). *Mean number of radical prostatectomy procedures, per hospital, per year.

We observed a negative statistically significant correlation between the hospital volume and the length of stay (r = − 0.303; p = 0.041), concerning the treatment with RP in the last three years (2018–2020) (Fig. 2).

## Discussion

A steep increase in the number of patients treated for localized prostate cancer in the Portuguese National Health Service has been observed in recent years. However, many hospitals have a very low case volume for RP and BT, and there are regional differences in the use of the RP, eRT and BT. Our results confirm that in Portugal, hospitals with higher case volume present lower length of stay for patients treated with RP.

The growing number of prostate cancer patients treated for localized disease in Portugal provably reflects the increase in the incidence and an earlier cancer diagnosis. The last prostate cancer incidence estimates in Portugal showed an average increase of 1.8%/year in the period 1998–2009^8^, which can be explained by the population aging due to a higher life expectancy^9^ and the higher use of PSA as a screening test^10^. The rise of screening procedures as well as the better access to healthcare (especially hospital consultations)^11^ may also constitute the determinants of the observed earlier cancer diagnoses, as already identified in other countries^12,13^. This contribute to patients being diagnosed at younger ages^14^ and at earlier stages^15^, further increasing the pool of localized prostate cancer patients.

Because of the increased treatments performed, the trends observed are also associated with an increase in the treatment cost. Considering the period with data for all the treatments (2007–2012) and the mean values used by the National Health System to reimburse the hospitals for each RP (1500€), BT (6407€) and eRT (3811€) treatment^16^ we observed an increase of the direct costs of approximately more than 2,700,000€ (39% increase within those 5 years).

International reports^17–24^ assessing the trends of RP within the first decade of the XXI century revealed an increasing frequency, with estimated increments over a 5-year period varying between 35 and 208%. It was the treatment modality chosen for 13% to 51% of the patients with localized prostate cancer. Data published after 2010 revealed a different picture such as in USA^24–26^ and Denmark^22^, where the number of RP slowly decreased after 2010–2011. A possible explanation was the combined effects of increasing use of deferred treatment^17,27^, improvements in eRT and increasing access to it and the decreased use of PSA screening after an evidence statement published by the U.S. Preventive Services Task Force in 2011, recommending against PSA based screening^28^. In Portugal, according to our results, we also observed an increase until 2011, but there was no decrease after 2011, possibly due to deferred treatments have not been so popularized as in other countries.

Fewer studies are available describing the trends of BT use. In the USA the number of treatments increased until 2002 and then decreased gradually^24,29,30^. In the Netherlands^31^ its frequency is still increasing and in the UK the number of treatments has remained stable^23^.

We observed a trend of increasing eRT use in Portugal. Data from the UK between 2000 and 2006 and from Japan in 2010^23,32^ have also showed an increase of eRT usage for primary prostate cancer treatment. Oppositely, the frequency of eRT treatments has been stable in the Netherlands^31^ and has been declining in the USA^27^. Other studies reported the increased use of adjuvant eRT^33,34^ and salvage RT^33^.

In 2020 there was a decrease in the number of procedures performed in comparison with 2019 (20% for RP and 39% for BT) which should be an indirect consequence of the COVID-19 pandemic due to an higher focus of the health services in the treatment of the COVID-19 patients. These observations contrasts with the Swedish^35^, German^36^ and Italian^37^ results where in 2020 there was a decreased in the number of prostate cancer diagnosis compared with 2019 but not radical treatments. One reason might be that in Portugal, despite the number of RP procedures stabilized after 2011, there was a peak in 2019, amplifying this apparent reduction from 2019 to 2020.

In our study we observed that 83% and 56% of the hospitals performed RP and BT, respectively, with a case-volume lower than the recommended by the expert group of the European School of Oncology. This fact may rise concerns about the expertise of the institutions and their health professionals delivering those treatments and shows that there is margin for increased concentration of prostate cancer treatments. Indeed, we observed, within the period 2018 to 2020, that in Portuguese hospitals a higher case volume of RP was associated with a lower length of stay (used as a proxy of better surgical outcomes). These results are in accordance with well-established knowledge, that had already demonstrated that higher-volume hospitals are associated with better outcomes including reduced mortality, morbidity, postoperative complications, length of stay, readmission, and cost-associated factors^3,4^.

This study has several strengths: The nationwide nature of our study, including all patients treated in national public hospitals; all the codification procedures were done by medical doctors with specific training, and verified by internal and external audits, first study of its kind in Portugal; as far as authors know, it is the longest time-trend analysis (20 years) of RP and BT internationally. However, this study has some limitations associated with using secondary data: possible codification errors of the performed treatments, though this should be residual; Information regarding the surgeons’ caseload within each institution and data on the patients’ comorbidities, details of prostate cancer disease and the setting of the performed treatments (primary, adjuvant or salvage) are lacking. The latter poses special limitations within the interpretation of temporal trends of eRT since we were unable to distinguish primary eRT from the adjuvant or salvage treatments. Also we cannot differentiate patients treated individually with eRT and BT from patients treated with a combination of eRT + BT. As far as the authors know the combination treatment is very rarely used in portuguese public hospitals. We were also unable to obtain data from the treatments performed in private hospital units turning our results valid only for public hospitals, as private medicine has different constrains that may affect the choice of treatment. This study also lacks information about the number of patients treated primarily only with hormonotherapy, experimental treatments or just followed in deferred treatment protocols. Although there is not published data, the authors believe that these should be residual in Portugal. In conclusion prostate cancer treatments for localized disease have increased significantly in Portugal in the last decades, but due to them being performed in several hospitals, the case volume per hospital is low for BT and particularly for RP. Our study confirms that higher case volume is associated with lower length of stay for patients treated with RP, adding arguments for the concentration in less hospitals of the treatments for these patients.

## Methods

The Portuguese National Health System is a universal system, tendentially free of charge for the users. The total number of hospitals were very high and in the last 20 years they have been aggregate into bigger hospitals.

The present study analyses data from the national database of diagnosis-related group (DRG) of the Portuguese Central Administration of the Health System (*Administração Central do Sistema de Saúde (ACSS)*), which is the public entity responsible for the registry of all the episodes of medical care of all hospitals belonging to the Portuguese National Health System. Each episode was coded and grouped in DRGs by a medical doctor that had a competence in clinical codification, using the International Classification of Diseases, Ninth Revision, Clinical Modification (ICD-9-CM) or, after 2017, using the International Classification of Diseases, Tenth Revision (ICD-10).

### Data collection

Data was provided to this research project upon written request and respective authorization from the authorities.

The annual number of RP, BT and eRT treatments among prostate cancer patients were assessed for each hospital, in all hospitals they were performed. Data were extracted by searching, among patients with prostate cancer as principal diagnosis (ICD-9-CM code 185; ICD-10 code C61) and: (a) RP code (ICD-9-CM code: 60.5 or ICD-10 codes: 0VT00ZZ or 0VT04ZZ, respectively for open and laparoscopic/robotic surgery); (b) BT code (ICD-9-CM code: 92.20, 92.27, 92.28 or 92.29, or ICD-10 codes 0VH031Z or DV10***); or (c) eRT code (ICD-9-CM DRG code 409 or ICD-10 codes DV00***, DV20*** or DVY0***). For eRT, only complete treatments, rather than sessions, were considered, and treatments with less than 20 sessions were excluded, since these are likely to correspond to palliative care.

Data on RP and BT were available for the period 2000–2020 but eRT data could only be obtained for 2007–2012.

Data codified with ICD-9-CM was obtained directly from the DRGs database of the ACSS. Due to a modification of the system, the data codified with ICD-10 codes was retrieved using a software (*Bilhete de Identidade da Morbilidade Hospitalar*) that generates reports from the same DRGs database.

### Data analysis

The annual number of patients treated with RP, BT or eRT in all hospitals is presented for the periods with available data. Trends analyses were carried out using the Joinpoint software, v.4.8.0.1.^19^. Joinpoint regression is a linear modelling approach that adjusts number of treatments (the dependent variables) for several years (the independent variable), to identify significant changes in the trends in each population. A Bayesian approach of joinpoint regression was used for modelling statistical data to identify the points in the trend where significant changes occur. For each of the segments obtained in the best model, the estimated Annual Percent Change (APC) was computed by fitting a regression line to the natural logarithm of the rates using the calendar year as a regressor variable. Afterwards, we find the best-fit model for each possible number of joinpoints, using permutation tests to determine the maximum number of joinpoints by testing the hypothesis that adding more joinpoints does not significantly improve model fit. The final Joint Point analysis graph was computed using Microsoft® Excel® for Microsoft 365 MSO (version 2308 Build 16. 0. 16,731. 20,182) 64-bit^38^ using data imported from final Joinpoint outputs analyses^38^.

For the most recent three years with data available we report on the average annual number of each procedure per hospital. The latter was also compared by type of hospital:—hospitals group IV-a;—hospitals group III;—hospitals group I and II^39^^39^(corresponding to oncology hospitals, generic central university hospitals, and other generic hospitals, respectively). Regions were compared for the last year (2012) with data available for the three procedures; for this purpose, the hospitals were grouped in three geographical areas (North, Center and South), that constitute the areas for inter-hospital patterns of patients’ referral. The association between hospital volume and length of stay for the last three years (2018–2020) was evaluated with a scatter plot and respective Pearson bivariate correlation coefficient. Statistics and the scatter plot were made using IBM® SPSS® Statistics version 26^40^. A p-value < 0.05 was considered as statistically significant, and the Confidence Interval (CI) used was 95%.

### Ethical aspects

The national database of DRG is registered in the National Commission of Data Protection and is authorized to provide anonymous data for research studies. No personal information or patient identifiable data was obtained or used for this study at any stage. Hospital specific data is published anonymously according to the rules of the database. This study protocol was approved and individual informed consent was waived by the Ethics Commission of Minho University (Comissão de Ética para a Investigação em Ciências da Vida e da Saúde da Universidade do Minho (CEICVS 039/2019). All methods were performed in accordance with the relevant guidelines and regulations.

### Supplementary Information

Supplementary Information.

## Data Availability

The datasets generated during and/or analyzed during the current study are available from the corresponding author on reasonable request, after ACSS authorization.
