# Supplementary material for: Prostate cancer treatment in Portugal: a nationwide analysis
Source: Sci Rep. 2023 Nov 8;13:19362. doi: 10.1038/s41598-023-46591-1 (PMC10632360; doi:10.1038/s41598-023-46591-1)
Supplement: Supplementary file 1 — Supplementary Information. [file 41598_2023_46591_MOESM1_ESM.docx]

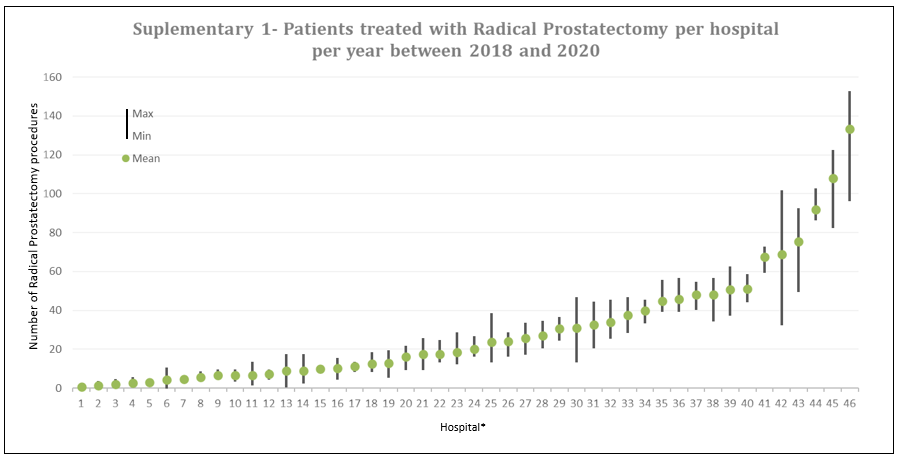


Max: maximum number of radical prostatectomy procedures, per hospital, per year, in the 2018-2020 period; Min – minimum number of radical prostatectomy procedures, per hospital, per year, in the 2018-2020 period; Mean: mean number of radical prostatectomy procedures, per hospital, per year, in the 2018-2020 period; * - each number identifies a specific hospital (hospital numbers were assigned in ascending order of mean radical prostatectomy procedures)


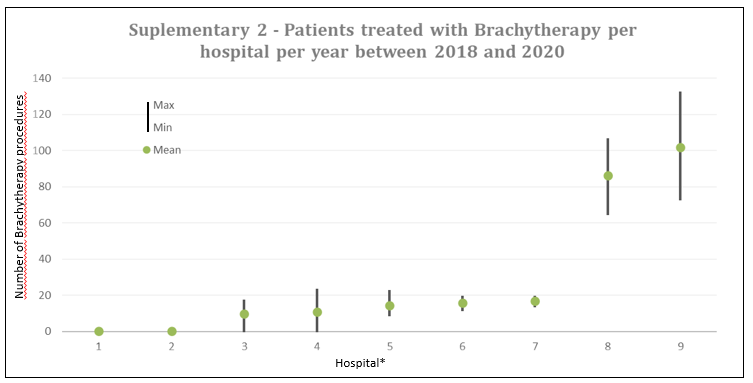


Max: maximum number of brachytherapies procedures, per hospital, per year, in the 2018-2020 period; Min – minimum number of brachytherapies procedures, per hospital, per year, in the 2018-2020 period; Mean: mean number of brachytherapies procedures, per hospital, per year, in the 2018-2020 period; * - each number identifies a specific hospital (hospital numbers were assigned in ascending order of mean brachytherapy procedures)


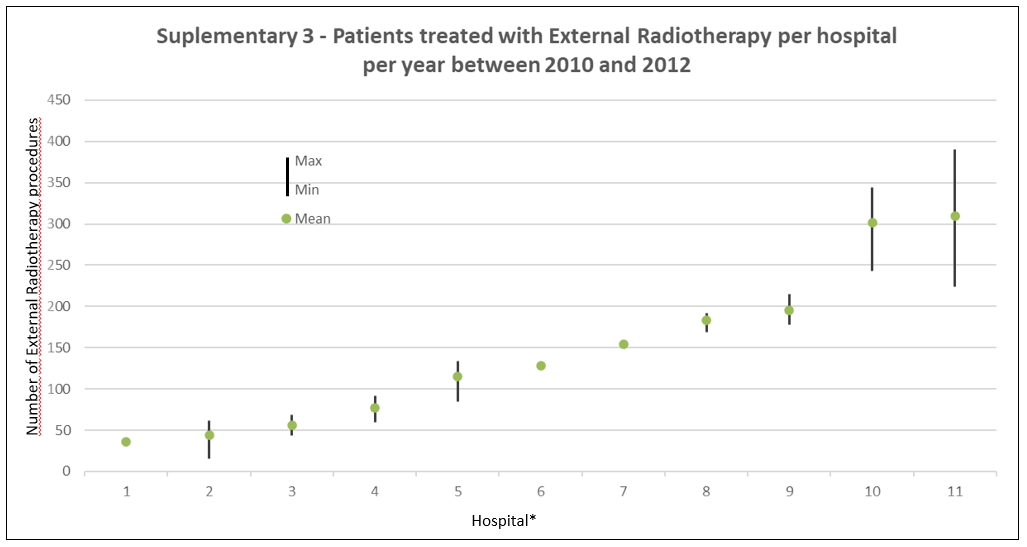


Max: maximum number of external radiotherapy procedures, per hospital, per year, in the 2010-2012 period; Min – minimum number of external radiotherapy procedures, per hospital, per year, in the 2010-2012 period; Mean: mean number of external radiotherapy procedures, per hospital, per year, in the 2010-2012 period; * - each number identifies a specific hospital (hospital numbers were assigned in ascending order of mean external radiotherapy procedures)
